# Right Hemiplegia Following Acute Carbon Monoxide Poisoning

**DOI:** 10.7759/cureus.16738

**Published:** 2021-07-29

**Authors:** Kenji Aoshima, Hidenaru Yamaoka, Shunsuke Nakamura, Tsuyoshi Nojima, Hiromichi Naito, Atsunori Nakao

**Affiliations:** 1 Department of Internal Medicine, Okayama Rōsai Hospital, Okayama, JPN; 2 Center for Graduate Medical Education, Okayama University Hospital, Okayama, JPN; 3 Department of Cardiology, Okayama Rōsai Hospital, Okayama, JPN; 4 Department of Emergency, Critical Care and Disaster Medicine, Okayama University Graduate School of Medicine, Dentistry and Pharmaceutical Sciences, Okayama, JPN

**Keywords:** carbon monoxide, carbon monoxide poisoning, hemiplegia, stroke, tia, globus pallidus lesions, neurologic manifestation

## Abstract

Acute carbon monoxide (CO) poisoning remains a common cause of poison-related death and influences neurological function. An 83-year-old female was transferred to our emergency unit due to hypertension with dizziness, headache, and right hemiplegia. There was no radiographic evidence of ischemic stroke. The family members reported that the patient may have been exposed to CO by briquettes burned inside a closed room. High flow oxygen therapy was given for suspected CO intoxication and her symptoms quickly improved. Although we do not have clear evidence, we presume that hemiplegia in our patient was caused by CO intoxication, based on rapid recovery with oxygen therapy, carboxyhemoglobin (COHb) level elevation (3.0%), polycythemia, and neuroimaging. Despite the hematogenous effects of CO, paralysis appeared to be more severe on her right side than on her left side. MRI and blood tests helped to support CO as the suspected cause of her hemiplegia. This case reconfirms the importance of medical interviewing by medical practitioners, even in an emergency setting.

## Introduction

Carbon monoxide (CO) poisoning can cause various neurological complications including movement disorders and mental deterioration through hypoxic brain injury. However, hemiplegia associated with CO intoxication is very rare, while peripheral neuropathy of the lower extremities is a known complication of CO poisoning [1].

Here, we report a hemiplegia case presumably caused by acute CO intoxication with chronic CO exposure followed by immediate improvement of the neurological symptoms with oxygen therapy. Although we do not have clear evidence, we believe our high index of suspicion that our patient’s hemiplegia was caused by CO intoxication is reasonable based on rapid recovery with oxygen therapy, polycythemia, and neuroimaging. Our report may serve as a reminder to clinicians to have a high degree of suspicion under the circumstances of CO exposure, although this is challenging for emergency physicians. Possible mechanisms of acute and delayed CO toxicity and suggested treatments are discussed.

## Case presentation

**Figure 1:**
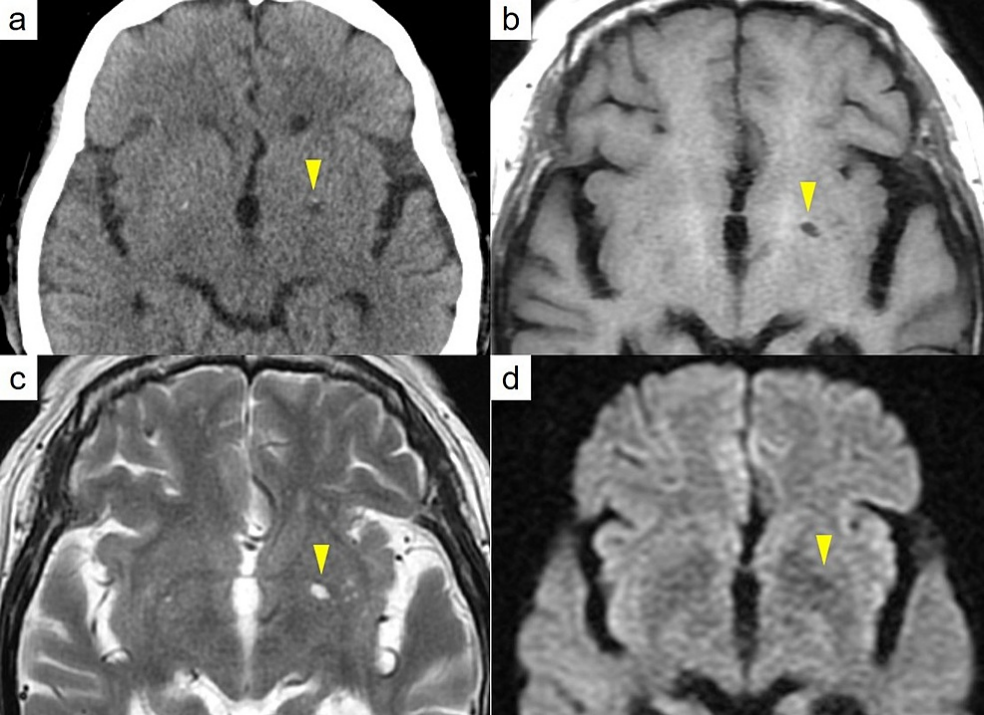
Radiographic images. a. Head CT showed no brain hemorrhage, but hypo-densities on the left globus pallidus. b. Head MRI: On T1 imaging, hypo-intensities on the left globus pallidus. c. On T2 imaging, hyper-intensities on the left globus pallidus. d. On  Diffusion-weighted imaging, hypo-intensities on the left globus pallidus.

A general physician referred an 83-year-old woman with right hemiplegia of the upper/lower limbs, hypertension, and headache to our ED. The patient had no initial loss of consciousness. She was medicated for hypertension and hyperlipidemia, and her blood pressure had been high over the previous few months. There was no episode of convulsion. The patient had no history of smoking or drug or alcohol abuse. Her symptoms worsened during transfer, and nausea and vomiting were noticed in the ambulance. On arrival at our ED, her blood pressure was 213/109 mmHg, her heart rate was 73 bpm, and her SpO2 was 100% in room air. Laboratory blood tests results were as follows: RBC count, 516 x 104/μl; hemoglobin, 15.7 g/dl; sodium, 143 mmol/L; potassium, 4.0 mmol/L; calcium, 1.21mmol/L; blood sugar, 114 mg/dL; and D-dimer, 1.4 μg/mL. Blood gas analysis results obtained three hours after leaving her house revealed the following levels: PO2, 78.3 torr; PCO2, 30.1 torr; pH, 7.518; base excess, 2.3 mmol/l; and carboxyhemoglobin (COHb), 3.0%. Electrocardiography showed normal sinus rhythm without atrial fibrillation. Her Glasgow Coma Score was 12 (E1/V5/M6) and her National Institutes of Health (NIH) Stroke Scale/Score was 14 with sagging of the left side of her mouth, right hemiplegia of her upper/lower limbs, and numbness of her right upper limb. The patient’s left extremities were weak but still moved. Contrast-enhanced CT did not show any abnormalities and did not show occlusion or dissection of the internal carotid arteries. MRI demonstrated hyperintensity on T2/fluid-attenuated inversion recovery (FLAIR) and hypo-densities on CT/T1 on the left globus pallidus. Diffusion-weighted imaging (DWI) showed hypo-densities on the left globus pallidus (Figure 1). Magnetic resonance angiography revealed no sign of ischemia in the brain vessels.

On admission, the patient’s family member reported that she burns charcoal briquettes daily for house heating in a closed room. Based on the elevation of COHb levels to 3.0%, polycythemia, and an episode of possible chronic exposure to CO, the patient was suspected to have CO intoxication. Normobaric oxygen therapy was promptly started and her symptoms improved within an hour, associated with the return of her COHb level to within the normal range (0.6%). Since her clinical course was uneventful over the two-day hospitalization, she was discharged to outpatient follow-up. On one month follow-up, the patient claimed continuous dizziness, a possible symptom of delayed neurologic sequelae, which is a chronic symptom of CO poisoning.

## Discussion

CO is a colorless, odorless, highly toxic gas due to its strong affinity for hemoglobin, which is 200 to 250 times greater than that of oxygen. CO not only exposes tissues to a lack of oxygen but also inhibits the function of mitochondria [2, 3]. Thus, CO poisoning is suspected when blood gas test results show metabolic acidosis with an increased anion gap, in addition to an elevated COHb level and information about the scene, including ambient CO levels. Our patient’s COHb level was 3.0%, which was above the cut-off to diagnose CO poisoning without a smoking history. Most of the symptoms of CO poisoning are likely due to hypoxia and nonspecific, including mental and neurologic symptoms, nausea, malaise, and dizziness, which are similar to the symptoms of ischemia [1, 4, 5]. Neuroimaging plays an important role not only to rule out brain stroke but also to diagnose CO poisoning. Many reports have been published about bilateral hyperintensities in the globus pallidus on T2/FLAIR imaging in MRI, representing necrosis in the globus pallidus [6]. Two reasons for this change are mentioned: the globus pallidus can get easily damaged due to low blood supply to the anatomical issue, and it has the highest heme iron content, to which CO binds directly [7-9].

The clinical manifestation of hemiplegia seen in the present case may be explained by several mechanisms. CO intoxication may grossly impair neurons. Also, direct compression or venous obstruction by edema may lead to hemiplegia. Park EJ et al. indicated that catecholamine crises in the deep white matter and globus pallidus with CO intoxication are also seen with the Cushing reflex, which is associated with stroke [10, 11]. The clinical manifestation of CO intoxication is similar to that of transient ischemic attack (TIA), although the mechanisms of tissue hypoxia in those two pathologies are quite different. Lin CW et al. reported that the overall incidence of ischemic stroke was almost 2.5-fold greater in patients with CO poisoning than that in a control group [12]. These findings suggest that CO intoxication can not only mimic stroke but also trigger renal ischemia. Accordingly, victims of CO intoxication should be carefully followed up in the long term.

Immediate amelioration of symptoms after treatment with oxygen therapies may support our diagnosis. Environmental circumstances such as the patient’s daily burning of charcoal briquettes in a confined space and her family members’ similar episodes may also reasonably support our diagnosis. Also, her blood tests showed an elevated hemoglobin level and RBC count while the patient did not smoke or live at a high altitude. From this evidence, we assumed that CO intoxication rather than TIA likely triggered the hemiplegia. Arterial spin labeling perfusion imaging for CO intoxication may be considered as a diagnostic utility [13, 14].

## Conclusions

We experienced a rare hemiplegia case presumably caused by CO intoxication. Emergency clinicians should be aware of this unique etiology of hemiplegia. When a patient presents atypical neurological defects, our experience may remind clinicians of the importance of obtaining a detailed medical history regarding the patient’s environment, considering factors including CO inhalation.
